# Gout and dementia in the elderly: a cohort study of Medicare claims

**DOI:** 10.1186/s12877-018-0975-0

**Published:** 2018-11-14

**Authors:** Jasvinder A. Singh, John D. Cleveland

**Affiliations:** 1 Medicine Service, Birmingham VA Medical Center, 700 19th St S, Birmingham, AL 35233 USA; 2 Department of Medicine at School of Medicine, University of Alabama at Birmingham, 1720 Second Ave. South, Birmingham, AL 35294-0022 USA; 3 Division of Epidemiology at School of Public Health, University of Alabama at Birmingham, 1720 Second Ave. South, Birmingham, AL 35294-0022 USA; 4 University of Alabama at Birmingham, Faculty Office Tower 805B, 510 20th Street S, Birmingham, AL 35294-0022 USA

**Keywords:** Gout, Dementia, Risk, Older adults, Medicare, Claims database

## Abstract

**Background:**

Conflicting data in the literature raise the question whether gout, independent of its treatment, increases the risk of dementia in the elderly. Our objective was to assess whether gout in older adults is associated with the risk of incident dementia.

**Methods:**

We used the 5% Medicare claims data for this observational cohort study. We used multivariable-adjusted Cox proportional hazard models to assess the association of gout with a new diagnosis of dementia (incident dementia), adjusting for potential confounders/covariates including demographics (age, race, sex), comorbidities (Charlson-Romano comorbidity index), and medications commonly used for cardiac diseases (statins, beta-blockers, diuretics, and angiotensin converting enzyme (ACE)-inhibitors) and gout (allopurinol and febuxostat).

**Results:**

In our cohort of 1.71 million Medicare beneficiaries, 111,656 had incident dementia. The crude incidence rates of dementia in people without and with gout were 10.9 and 17.9 per 1000 person-years, respectively. In multivariable-adjusted analyses, gout was independently associated with a significantly higher hazard ratio of incident dementia, with a HR of 1.15 (95% CI, 1.12, 1.18); sensitivity analyses confirmed the main findings. Compared to age 65 to < 75 years, age 75 to < 85 and ≥ 85 years were associated with 3.5 and 7.8-fold higher hazards of dementia; hazards were also higher for females, black race or people with higher medical comorbidity.

**Conclusion:**

Gout was independently associated with a 15% higher risk of incident dementia in the elderly. Future studies need to understand the pathogenic pathways involved in this increased risk.

## Background

Gout, the most common inflammatory arthritis in the adults, is characterized by hyperuricemia, monosodium urate crystal formation and inflammation. Dementia, characterized by progressive deterioration of cognitive ability and function, is a common disease of the elderly that has replaced ischemic heart disease as the leading cause of death in England and Wales [1].

Dementia can be caused by Alzheimer’s disease (60–70%), vascular dementia (20%), and other conditions such as Parkinson’s disease, etc. [2]. Dementia is frequently diagnosed based on the clinical presentation. Various clinical tests used for the evaluation of cognitive function i.e. mini mental state examination (MMSE) [3], global deterioration scale (GDS) [4] etc., can document the severity of cognitive impairment quantitatively and allow the assessment of cognitive changes over time [5]. Diagnostic criteria for various dementia subtypes have been developed, including the NINDS-AIREN criteria [6], NINCDS-ADRDA criteria [7], etc. Additionally, the recognition of biomarkers for certain subtypes, such as Alzheimer’s disease including structural MRI, molecular neuroimaging with PET, and cerebrospinal fluid analyses are bridging the knowledge gap between pathophysiology and clinical manifestations [8, 9].

Dementia is associated with limitation of functional ability [10] and deficits in quality of life [11], which can lead to the loss of independence and increased morbidity and mortality [12, 13]. The number of people with dementia worldwide is expected to quadruple to 115 million by 2050 [14]. Therefore, dementia is a significant public health problem of increasing impact. Studies are needed to identify novel risk factors for dementia beyond demographics, cardiovascular disease and head injury [15]. Identification of novel risk factors can lead to back-to-the-bench translational studies and identify new pathways and mechanisms of dementia.

The pursuit of a link between gout/hyperuricemia and dementia has led to contradictory results. Most observational studies, including population-based studies, showed that hyperuricemia was associated with a higher risk of dementia and cognitive dysfunction [16–21], while a few studies found hyperuricemia to be associated with a lower risk of dementia [22, 23].

Recently, a large French population-based study in the elderly (65 years or older) showed that hyperuricemia was associated with a higher risk of dementia and with MRI changes of aging in the brain (extensive white matter hyperintensity volume; *p* = 0.10), providing the first clinical-pathological correlation to-date between hyperuricemia and brain changes [24]. One of the studies that found gout to be associated with a reduced risk of dementia found this risk reduction to be limited to treated gout patients (urate-lowering drugs or colchicine) and no association was found in untreated gout patients [23], indicating that protection against dementia risk in patients with gout may be medication-related. These observations raise the question whether gout, independent of its treatment, increases the risk of dementia in the elderly, which our study aimed to address. As a secondary study objective, we also assessed whether this risk varied by demographic features or the presence of common comorbidities.

## Methods

### Data sources and study sample

We used the 5% random Medicare claims sample from 2006 to 2012 for this cohort study. Data were obtained from the Centers for Medicare and Medicaid Services (CMS) data warehouse. We followed the with the Strengthening of Reporting in Observational studies in Epidemiology (STROBE) guideline for reporting the methods and results of our study. People were eligible for this study if they were enrolled in Medicare fee-for-service (Parts A, B) and not enrolled in a Medicare Advantage Plan from 2006 to 2012, and resided in the U.S. The study was approved by the Institutional Review Board (IRB) at the University of Alabama at Birmingham (UAB).

### Predictor of interest and covariates

Gout, confirmed by the existence of two claims at least 4-weeks apart with an International Classification of Diseases, ninth revision, common modification (ICD-9-CM) code of 274.xx, was our main predictor of interest. This definition has high accuracy with sensitivity of 90% and specificity of 100% [25]. People without gout at baseline were are the risk of being diagnosed with gout during the follow-up. The diagnosis of gout had to precede the diagnosis of dementia, i.e., we included all prevalent cases of gout at the beginning of study window and all new gout cases during study period, as long as the gout diagnosis preceded the diagnosis of dementia. We excluded people with only one claim for gout from the analysis.

We included several covariates and potential confounders. Demographic characteristics including age, sex and race were obtained from the Medicare beneficiary summary file. Comorbidity was assessed from all inpatient and outpatient claim files that included diagnosis codes and claim dates from Medicare part A and B files, and calculated using the Charlson-Romano index, which is a commonly used validated weighted comorbidity index, developed for the claims data [26]. Similarly, we used ICD-9-CM codes from all inpatient and outpatient claim files to identify hypertension, hyperlipidemia, and coronary artery disease. All demographics except age were assessed at baseline. Age was a time-varying covariate, which was allowed to change during the study follow-up. Common medications for the treatment of cardiovascular diseases (statins, beta-blockers, diuretics, and angiotensin converting enzyme (ACE)-inhibitors) and for urate-lowering in gout (allopurinol and febuxostat) were included, data derived from the Medicare part D file that contains all prescription claims (dose, supply, and drug name). Medication use variables were modeled as time-varying covariates, i.e., allowed to vary throughout the study period.

### Dependent variable/outcome of interest

The outcome of interest was incident dementia, identified by the occurrence of two claims at least 4-weeks apart with ICD-9-CM codes for 290.xx, 294.1x, or 331.2, using the same codes as the Quan-Charlson index, a validated medical comorbidity index, with no claims for dementia in the baseline period of at least 1 year (1/1/2005 to 12/31/2005). This approach is valid, with positive and negative predictive values of 96 and 98% and specificity of 100% [27].

### Statistical analyses

We used a person-day file, assessing risk of outcome each day of follow-up. The time origin for analyses was the time for the second claim of gout. The study follow-up for each person was censored at the time of the occurrence of incident dementia, end of the study period (12/31/2012) or death, whichever occurred first. Crude incidence rates were calculated per 1000 person-years for incident dementia in people with or without gout. Unadjusted characteristics were compared using a t-test or a chi-square test, as appropriate. We used a multivariable-adjusted Cox proportional hazard analysis to examine the association of gout with incident dementia, controlling for demographics, comorbidity and medication use (model 1). We calculated hazard ratios (HR) and 95% confidence intervals (CI). Sensitivity analyses substituted continuous Charlson-Romano index score with categorical score (model 2) or individual comorbidities plus hypertension, hyperlipidemia, and coronary artery disease (model 3).

## Results

There were 1,712,821 Medicare beneficiaries in our study cohort. Of these, 111,656 had incident dementia during the study follow-up. With 106,346 incident dementia cases in people without gout and 5310 dementia cases in people with gout, the crude incidence rates were 10.9 and 17.9 per 1000 person-years, respectively. The mean (SD) [median; interquartile range] time from the diagnosis of gout to the diagnosis of dementia was 2.3 years (1.7) [2.0; 0.9 to 3.5].

Compared to people who did not develop dementia, people who developed incident dementia were 5 years older, more likely to be female, Black, have higher Charlson-Romano score and higher prevalence of all Charlson-Romano comorbidities except AIDS (Table 1). Hypertension, hyperlipidemia and coronary artery disease were more frequent in people with dementia, compared to people without (Table 1).

**Table 1:** Demographic and clinical characteristics of beneficiaries who developed incident dementia^a^

	All beneficiaries	Incident Dementia^a^ during the follow-up		*p*-value
		No	Yes	
Total, N (beneficiaries)	1,712,821	1,601,165	111,656^b^	N/A
Age, mean (SD)	75.2 (7.5)	**74.9 (7.5)**	**80.0 (7.1)**	**< 0.0001**
Sex, N (%)				**< 0.0001**
Male	729,781 (42.6%)	**693,059 (43.3%)**	**36,722 (32.9%)**	
Female	983,040 (57.4%)	**908,106 (56.7%)**	**74,934 (67.1%)**	
Race/Ethnicity, N (%)				**< 0.0001**
White	1,476,044 (86.2%)	**1,380,894 (86.2%)**	**95,150 (85.2%)**	
Black	139,833 (8.2%)	**128,824 (8.0%)**	**11,009 (9.9%)**	
Other/unknown	96,944 (5.7%)	**91,447 (5.7%)**	**5497 (4.9%)**	
Charlson-Romano score, mean (SD)	1.56 (2.36)	**1.52 (2.34)**	**2.18 (2.49)**	**< 0.0001**
Charlson-Romano score				**< 0.0001**
0	912,029 (53.2%)	**870,890 (54.4%)**	**41,139 (36.8%)**	
1	174,091 (10.2%)	**159,811 (10.0%)**	**14,280 (12.8%)**	
≥ 2	626,701 (36.6%)	**570,464 (35.6%)**	**56,237 (50.4%)**	
Charlson-Romano comorbidities				
Myocardial Infarction	67,609 (3.9%)	**62,029 (3.9%)**	**5580 (5.0%)**	**< 0.0001**
Heart Failure	198,607 (11.6%)	**180,109 (11.2%)**	**18,498 (16.6%)**	**< 0.0001**
Peripheral vascular disease	164,078 (9.6%)	**147,448 (9.2%)**	**16,630 (14.9%)**	**< 0.0001**
Cerebrovascular disease	162,203 (9.5%)	**142,965 (8.9%)**	**19,238 (17.2%)**	**< 0.0001**
Dementia	58,582 (3.4%)	**47,204 (2.9%)**	**11,378 (10.2%)**	**< 0.0001**
Chronic pulmonary disease	266,474 (15.6%)	**245,787 (15.4%)**	**20,687 (18.5%)**	**< 0.0001**
Connective tissue disease	47,610 (2.8%)	**43,566 (2.7%)**	**4044 (3.6%)**	**< 0.0001**
Peptic ulcer disease	32,015 (1.9%)	**28,853 (1.8%)**	**3162 (2.8%)**	**< 0.0001**
Mild liver disease	8437 (0.49%)	7918 (0.49%)	519 (0.46%)	0.17
Diabetes	315,236 (18.4%)	**289,481 (18.1%)**	**25,755 (23.1%)**	**< 0.0001**
Diabetes with end organ damage	92,786 (5.4%)	**84,100 (5.3%)**	**8686 (7.8%)**	**< 0.0001**
Hemiplegia	13,668 (0.80%)	**11,972 (0.75%)**	**1696 (1.5%)**	**< 0.0001**
Renal failure/disease	58,438 (3.4%)	**53,802 (3.4%)**	**4636 (4.2%)**	**< 0.0001**
Any tumor, leukemia, or lymphoma	172,705 (10.1%)	**160,676 (10.0%)**	**12,029 (10.8%)**	**< 0.0001**
Moderate or severe liver disease	1969 (0.11%)	1852 (0.12%)	117 (0.10%)	0.30
Metastatic cancer	17,879 (1.0%)	**17,113 (1.1%)**	**766 (0.69%)**	**< 0.0001**
AIDS	546 (0.03%)	519 (0.03%)	27 (0.02%)	0.14
Hypertension	823,180 (48.1%)	**754,449 (47.1%)**	**68,731 (61.6%)**	**< 0.0001**
Hyperlipidemia	597,299 (34.9%)	**554,334 (34.6%)**	**42,965 (38.5%)**	**< 0.0001**
Coronary artery disease	299,331 (17.5%)	**273,719 (17.1%)**	**25,612 (22.9%)**	**< 0.0001**

*SD* standard deviation, *N/A* not applicable

Bold represents statistical significance, with a *P*-value < 0.05

^a^Incident dementia defined as the occurrence of two claims at least four-weeks apart with ICD-9-CM codes, 290.xx, 294.1x, or 331.2, with baseline 365 days without any claim for dementia

^b^Met eligibility criteria and did not have dementia in the baseline 365-day period

In multivariable-adjusted analyses, gout was independently associated with a significantly higher hazard ratio of incident dementia, with a HR of 1.15 (95% CI, 1.12, 1.18), an association that persisted in sensitivity analyses (Table 2). Compared to age 65 to < 75 years, older age groups were associated with 3.5 and 7.8-fold higher hazards of dementia; hazards were also higher for females, Black race or people with higher medical comorbidity (Table 2).

**Table 2:** Association of gout and other potential risk factors with incident dementia in adults 65 years or older

	Multivariable-adjusted (Model 1)^a^		Multivariable-adjusted (Model 2)^a^		Multivariable-adjusted (Model 3)^a^	
	HR (95% CI)	*P*-value	HR (95% CI)	*P*-value	HR (95% CI)	*P*-value
Age (in years)						
65 - < 75	Ref		Ref		Ref	
75 - < 85	**3.53 (3.48, 3.58)**	**< 0.0001**	**3.49 (3.44, 3.54)**	**< 0.0001**	**3.35 (3.30, 3.40)**	**< 0.0001**
≥ 85	**7.81 (7.68, 7.95)**	**< 0.0001**	**7.75 (7.62, 7.88)**	**< 0.0001**	**6.80 (6.68, 6.92)**	**< 0.0001**
Sex						
Male	Ref		Ref		Ref	
Female	**1.34 (1.32, 1.36)**	**< 0.0001**	**1.33 (1.31, 1.34)**	**< 0.0001**	**1.26 (1.25, 1.28)**	**< 0.0001**
Race						
White	Ref		Ref		Ref	
Black	**1.29 (1.26, 1.32)**	**< 0.0001**	**1.33 (1.30, 1.35)**	**< 0.0001**	**1.24 (1.22, 1.27)**	**< 0.0001**
Other	**0.87 (0.85, 0.89)**	**< 0.0001**	**0.89 (0.87, 0.92)**	**< 0.0001**	**0.85 (0.82, 0.87)**	**< 0.0001**
Charlson-Romano score, per unit change	**1.16 (1.15, 1.16)**	**< 0.0001**	N/A		N/A	
Charlson-Romano score						
0	N/A		Ref		N/A	
1			**1.72 (1.68, 1.75)**	**< 0.0001**		
≥ 2			**2.17 (2.14, 2.19)**	**< 0.0001**		
Gout	**1.15 (1.12, 1.18)**	**< 0.0001**	**1.17 (1.14, 1.20)**	**< 0.0001**	**1.17 (1.14, 1.21)**	**< 0.0001**

*N/A* not applicable, *HR* hazard ratio, *CI* confidence interval, *Ref* referent category

Bold represents statistical significance, with a *P*-value < 0.05

^a^Model 1 included Charlson-Romano score as a continuous variable; Model 2 replaced it with categorized Charlson-Romano score; and Model 3 replaced it with each of the 17 Charlson-Romano comorbidities plus hypertension, hyperlipidemia, and coronary artery disease. All models were also adjusted for medications for cardiovascular diseases (statins, beta-blockers, diuretics, ACE-inhibitors) and for urate-lowering therapies for gout (allopurinol, febuxostat)

**Fig. 1:**
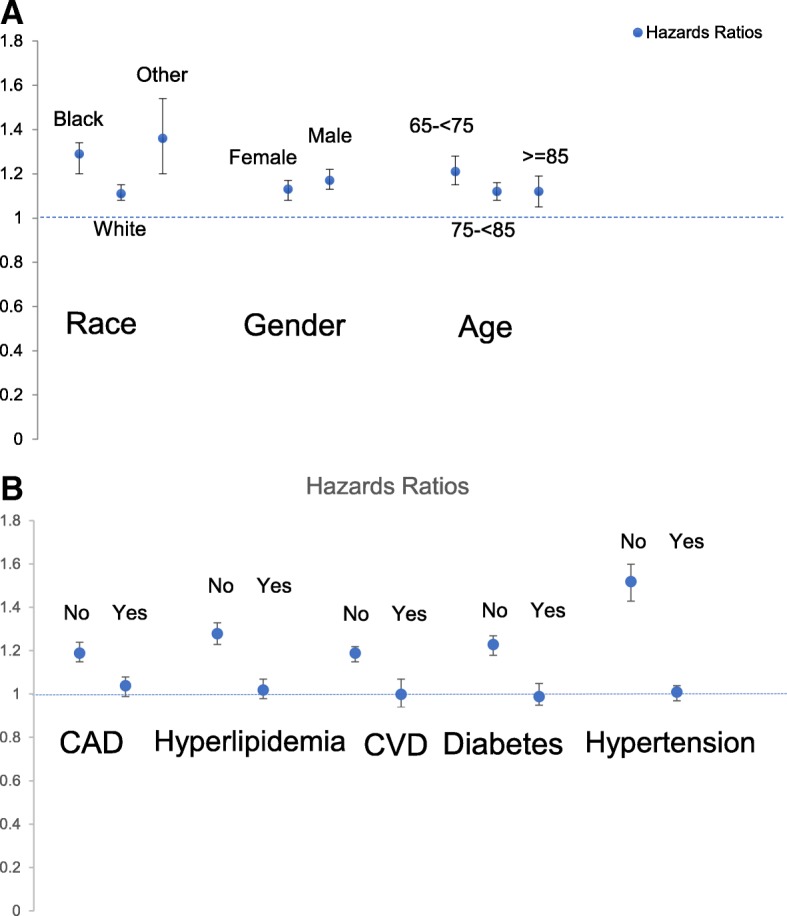
Association of gout with incident dementia by subgroups- Race, sex and age (**a**); presence/absence of CAD, hyperlipidemia, CVD, diabetes and hypertension (**b**). Point estimates are indicated by filled circles and the whiskers represent the 95% confidence intervals. Hazards ratio are statistically non-significant when the confidence interval includes the null hazard ratio of 1.0. For example, none of the associations of gout with incident dementia were significant in the presence of key comorbidities (CAD, hyperlipidemia, CVD, diabetes and hypertension) except borderline significance in the presence of CAD. *P*-values for interaction terms were as follows: Gout*age, *p* < 0.0001; Sex*gout, *p* = 0.0018; Gout*race, *p* = 0.035; Gout*CAD *p* < 0.0001; Gout*hyperlipidemia *p* < 0.0001; Gout*CVD *p* < 0.0001; Gout*diabetes *p* < 0.0001; Gout*hypertension *p* < 0.0001

Subgroup analyses indicated that there were minor differences by race, age and sex (Fig. 1), but all hazard ratios between gout and incident dementia were significant across categories of these variables. Gout was significantly associated with dementia in patients without key comorbidities (CAD, hyperlipidemia, CVD, diabetes, hypertension) with HR ranging 1.2–1.5, but not in patients with each of these comorbidities, except a borderline association in those with CAD, with HR of 1.07 (95% CI, 1.01 to 1.14; Fig. 1).

## Discussion

In this study, we found that elderly people with gout had a 15–18% higher hazard of dementia, compared to people without gout, after adjusting for effects of age, sex, race, medical comorbidities, and common medications for cardiac diseases and gout. As expected this association was statistically significant in this study with a large sample size, but the relative increase in risk was small. On the other hand, the population of Americans 65 years or older is predicted to grow from 34.4 million in 2000 to > 70 million in 2030 [28]. Given the significant associated morbidity and mortality of dementia in this age group and its common occurrence in this age group [1, 29–31], even this small increase in dementia risk makes this finding clinically relevant. Older age was a strong risk factor for dementia, although female sex, Black race and higher comorbidity were also significantly associated with higher risk of dementia.

A higher risk of dementia in people with gout should not be surprising, since gout is associated with hyperuricemia, chronic inflammation and oxidative stress, and several or all of these mechanisms may play a key role in the pathogenesis of dementia. Recently noted positive association of hyperuricemia with MRI lesions, extensive white matter hyperintensity volume (a biomarker of small vessel disease; *p* = 0.10) in elderly people with gout [24], is consistent with our finding of higher risk of dementia in the elderly with gout. Evidence links oxidative stress to neurodegenerative processes in dementia is accumulating [32]. Animal studies show that associated oxidative damage in brain precedes Aß-amyloid deposition and neuronal injury [33]. The conversion of hypoxanthine into uric acid by xanthine oxido-reductase (XOR) system involves two steps and leads to the formation of superoxide species that increases oxidative stress. Thus, oxidative stress may be the final common pathway in both gout and dementia, that can at least partially explain the increased risk of dementia in elderly people with gout. A better understanding of pathways that lead to higher risk of dementia in people with gout will not only improve our understanding of this association between two common conditions in the elderly population, but may also pave the path for discovery of new treatments targeting these pathways to prevent or delay the onset of dementia. The elderly population in the U.S. that is 65 years or older is predicted to grow from 34.4 million in 2000 to > 70 million in 2030, indicating that studies of potential risk factors of common public health problems in this population, such as dementia, are needed.

Additionally, research has shown that uric acid has both a pro-oxidant action [34, 35] and an anti-oxidant action [36, 37]. While the former is potentially neurotoxic, the latter may make uric acid potentially neuroprotective. Some have proposed that hyperuricemia is associated with elevated total serum antioxidant capacity among individuals with atherosclerosis, indicating that hyperuricemia may be a compensatory mechanism to counteract oxidative damage related to atherosclerosis and aging in humans [38]. However, most evidence points to hyperuricemia being a risk factor for cardiovascular disease [39–42]. It is possible that the association of hyperuricemia with cardiovascular disease is due to concomitant oxidative stress (a hypothesis that remains to be proven), which is implicated in the pathogenesis of dementia [32, 43, 44], or due to its association with other cardiovascular disease risk factors [45].

We found that gout was associated with a significantly increased risk of dementia in people without each of the common comorbidities, CAD, hyperlipidemia, CVD, diabetes, or hypertension. On the other hand, gout was not associated with dementia risk in in the presence of any of these comorbidities. This is an interesting finding from the subgroup analyses and novel, to our knowledge. Whether this indicates that the key pathways of disease causation in dementia differ based on the presence/absence of these conditions or that gout only imparts the dementia risk in the absence of these conditions, remains to be seen. This finding needs to be reproduced in other elderly cohorts.

Our study has several limitations that must be considered while interpreting findings. Our findings are from a representative elderly population, 65 years or older, and therefore, findings should not be generalized to younger populations. Several limitations are related to claims data we used. Despite the use of a valid diagnostic code algorithm to identify people with dementia, our study findings are at the risk of non-differential misclassification bias, since the measurement error in dementia diagnosis (study outcome) is unlikely to have differed by the exposure (gout) status. This may have biased the results towards the null, making these results conservative estimates. Dementia diagnosis was not based on diagnostic criteria, MMSE scores, or magnetic resonance imaging (MRI) characteristics, since these data are not available in Medicare claims. Availability of such data could have allowed additional insights into the observed association and possibly reduced the misclassification bias. Due to an observational study design, residual confounding is possible; we included several potential confounders and covariates to reduce the confounding bias. A delay or failure of diagnosis of dementia may have occured if people with symptoms of dementia were late or fail to seek medical help. A surveillance bias in people diagnosed with gout for other chronic conditions such as dementia may have led to a higher rate of the diagnosis of dementia to some extent. However, there are no previous known associations of gout with dementia and on the contrary some studies previously showed hyperuricemia (a cardinal feature of gout) to be associated with a lower risk of dementia [22, 23], making this surveillance bias unlikely. Medicare data does not include body mass index data, which limited our ability to account for the role that obesity may play in this relationship, or whether general versus abdominal obesity differs in their effect on the noted association. The lack of the availability of laboratory data in the Medicare claims limited our ability to further investigate whether the noted association was mediated by hyperuricemia, chronic inflammation and/or oxidative stress associated with gout. This question needs further study with different study design and data source. A study of dementia subtypes was outside the scope of this study, and needs to be addressed with future studies. Study strengths included a large sample size, robustness of findings in sensitivity analyses and use of a U.S. representative population of the elderly.

## Conclusions

In conclusion, we found that gout was independently associated with a 15–18% higher hazard of dementia in the elderly. This increased risk might be due to the effects of hyperuricemia, oxidative stress, and/or inflammation. Future studies should also explore whether interventions targeting these can ameliorate this increased risk.

## Abbreviations

ACE inhibitor: Angiotensin converting enzyme inhibitor
CMS: Centers for Medicare and Medicaid Services
ICD-9-CM: International Classification of Diseases, ninth revision, common modification
IRB: Institutional Review Board
STROBE: Strengthening of Reporting in Observational studies in Epidemiology
UAB: University of Alabama at Birmingham
